# The effects of aqueous extract of Maca on energy metabolism and immunoregulation

**DOI:** 10.1186/s40001-020-00420-7

**Published:** 2020-06-29

**Authors:** Wenting Fei, Yan Hou, Na Yue, Xue Zhou, Yujie Wang, Linyuan Wang, Aimin Li, Jianjun Zhang

**Affiliations:** 1 School of Traditional Chinese Medicine, Beijing University of Chinese Medicine, No. 11, East Road, North 3rd Ring Road, Chaoyang District, Beijing, 100029 China; 2 School of Chinese Materia Medica, Beijing University of Chinese Medicine, Sunshine South Street, Fangshan District, Beijing, 100102 China; 3 New Era Health Industry(Group) Co., Ltd, No. 10, Science Park Road, Changping District, Beijing, 102206 China

**Keywords:** Maca, Aqueous extract of Maca, Traditional Chinese medicine, Spleen, Energy metabolism, Immunoregulation

## Abstract

**Background:**

In the present work, we investigated the effects of aqueous extract of Maca (AEM) on energy metabolism and immunoregulation in spleen-deficient mice.

**Method:**

We established a cyclophosphamide-induced spleen-deficiency model with ginseng, a herb that strengthens splenic function, as a control. Sixty male Kunming mice were randomly divided among 5 groups: normal, model, ginseng control (1.5 g/kg), AEM high dose (1.5 g/kg), and AEM low dose (0.75 g/kg). All animals, except those in the normal group, were injected with cyclophosphamide to induce spleen deficiency. Furthermore, we investigated differences in the thermotropic behaviors of mice using the Animal Thermotropism Behavior Surveillance System to detect energy metabolism-related assays and immune regulation assays.

**Results:**

Mice given AEM exhibited tropism in response to hot plate exposure. AEM inhibited loss of body weight and immune organ atrophy caused by cyclophosphamide, increased the cAMP/cGMP ratio in blood, and enhanced the activities of Na^+^–K^+^-ATPase, Ca^2+^–Mg^2+^-ATPase, lactate dehydrogenase, and hepatic glycogen. AEM significantly reversed declining white blood cells and platelet counts, and increased the hemoglobin content within peripheral blood cells. AEM improved the protein levels of IFN-γ, TNF-β, IL-2, and IL-4 in the spleen.

**Conclusions:**

Maca possesses the Traditional Chinese Medicine (TCM) property of warm and appears to strengthen spleen function.

## Background

*Lepidium meyenii*, known as Maca, is traditionally consumed fresh or dehydrated after being boiled in water or milk [1, 2]. Maca is useful for treating sexual dysfunction, osteoporosis, benign prostatic hyperplasia, memory and learning, depression and anxiety [1, 3–7]. In China, it is considered as a Traditional Chinese Medicine (TCM) agent [8, 9]. However, its theoretical guidance of TCM agent is needed to further explore.

Scientists have investigated the endocrinological, neurological, and immunological outcomes associated with TCM, such as enhanced or weakened neurological functions, up- or down-regulated neurotransmitter molecules, and alterations to the ratio of cyclic adenosine monophosphate to cyclic guanosine monophosphate (cAMP/cGMP). An animal thermotropism behavior surveillance system has been developed and used to distinguish the hot and cold properties of medicinal herbs used in TCM [10]. Recently, a new theory set forth the belief that the four Qi (cold, cool, warm, and hot) of TCM are associated with energy metabolism, such as the activities of Na^+^–K^+^-ATPase, Ca^2+^–Mg^2+^-ATPase, and the levels of lactate dehydrogenase (LDH), and SDH. For example, TCM of ginseng is believed to exhibit “hot” herbal properties [11].

Spleen-deficiency syndrome (SDS) is often treated using TCM, and is characterized by poor appetite, fullness, sleepiness after eating, nausea, vomiting, fatigue, facial and tongue pallor, weight loss, and loose stools [12]. Cyclophosphamide (CYP) is the most commonly used antitumor drug in clinical practice. However, it has side effects, including immunosuppression, potentially eliciting leukopenia, loss of appetite, nausea, vomiting, and fatigue [11]. The clinical manifestations of immunosuppression are quite similar to SDS. Therefore, intraperitoneal injection of high-dose CYP can be used to establish a spleen-deficiency animal model [13, 14].

Immunomodulation is an important means of enhancing the body’s immunity to a variety of diseases, and is also very important for strengthening the spleen in TCM. Immunomodulation refers to nonspecific activation of the immune system, and implies a non-antigen-dependent stimulation of the activities of macrophages, natural killer (NK) cells, granulocytes, complement, and lymphocytes, and the production of effector molecules [15]. Cytokines exert a vast array of immunoregulatory actions critical to human biology and disease.

This research has studied the energy metabolism and immunoregulatory mechanisms of Maca. This is the first study to examine the TCM properties of Maca that are responsible for its popularization in China. We observed the effects of Maca on animal behaviors and functions to establish an objective and innovative method for determining and verifying the TCM properties of Maca to inform the development of new kinds of TCM resources.

## Materials and methods

### Materials and reagents

The approval was obtained from the ethics committee. Maca powder was purchased from New Era Health Industry Co. Ltd. (Beijing, China). Aqueous extract of Maca powder (AEM) was prepared according to traditional methods. The powder (50 g) was placed into a container with 1000 ml of water. The mixture was automatically stirred and extracted within a 70 °C water bath for 1.5 h, then centrifuged at 4000 R/min for 10 min. Afterward, the supernatant was collected, and the extraction was repeated twice. The two supernatants were combined, and 10% of the supernatant was concentrated and dried to achieve a paste ratio of 63.14%. The remaining 90% of the supernatant was concentrated and distilled water was added to a volume of 150 ml to obtain a raw drug concentration of 3000 mg/ml. The concentrate, containing 3000 mg/ml Maca, was placed in small vials and stored in a refrigerator at 4 °C for further use. Before the experiments, 3000 mg/ml Maca was dissolved in an aqueous solution to concentrations of 1500 and 750 mg/ml. Ginseng extract was obtained from Beijing Pharmaceutical Co., Ltd. The total content of ginsenosides Rg1 and Re was 0.55% and the ginsenosides Rb1 was 0.74%. Injectable CTX was purchased from Bioway Co. Ltd (Shanghai, China).

### Animals

Sixty Kunming male mice (18–22 g) were obtained from SPF Biotechnology Co. Ltd. (Beijing). The mice were housed on a 12 h light–dark cycle (23 ℃ ± 2 ℃; 60% ± 5% humidity). The mice were fed a standard diet and had free access to water. The animals acclimatized to their surroundings for 1 week. The trials were administered according to the Guide for the Care and Use of Laboratory Animals. The animal experiments were approved by the Ethics Committee in Beijing University of Chinese Medicine.

### Establishment of immunosuppressed mice model

The experimental groups were immunosuppressed by intraperitoneal injection of CYP (60 mg/kg/d) on days 12, 13, and 14 according to the previous reports. The normal group did not receive CYP injections. The animals were randomly divided into 5 groups, with 12 animals in each group. After the model establishment, the normal group and model group were treated once daily with physiological saline. The other groups received various orally administered solutions by gavage in mice, including 750 (AEM low dose group, AEM-L), 1500 mg/kg (AEM high-dose group, AEM-H) and 1.5 g/kg body weight in ginseng group as a positive control, once per day for 14 continuous days.

### Bodyweight and immune organ indices

Twenty-four hours after the final drug administration, the animals were weighed. Mice were placed under anesthesia using 3–5% isoflurane and then killed. The spleen and thymus were collected and immediately weighted to calculate the spleen and thymus indices according to the following formula:

$${\text{Index }}\left( {{\text{mg}}/{\text{g}}} \right) \, = {\text{ weight of thymus or spleen}}/{\text{ body weight}} .$$

### Thermotropism behaviors in mice

**Fig. 1:**
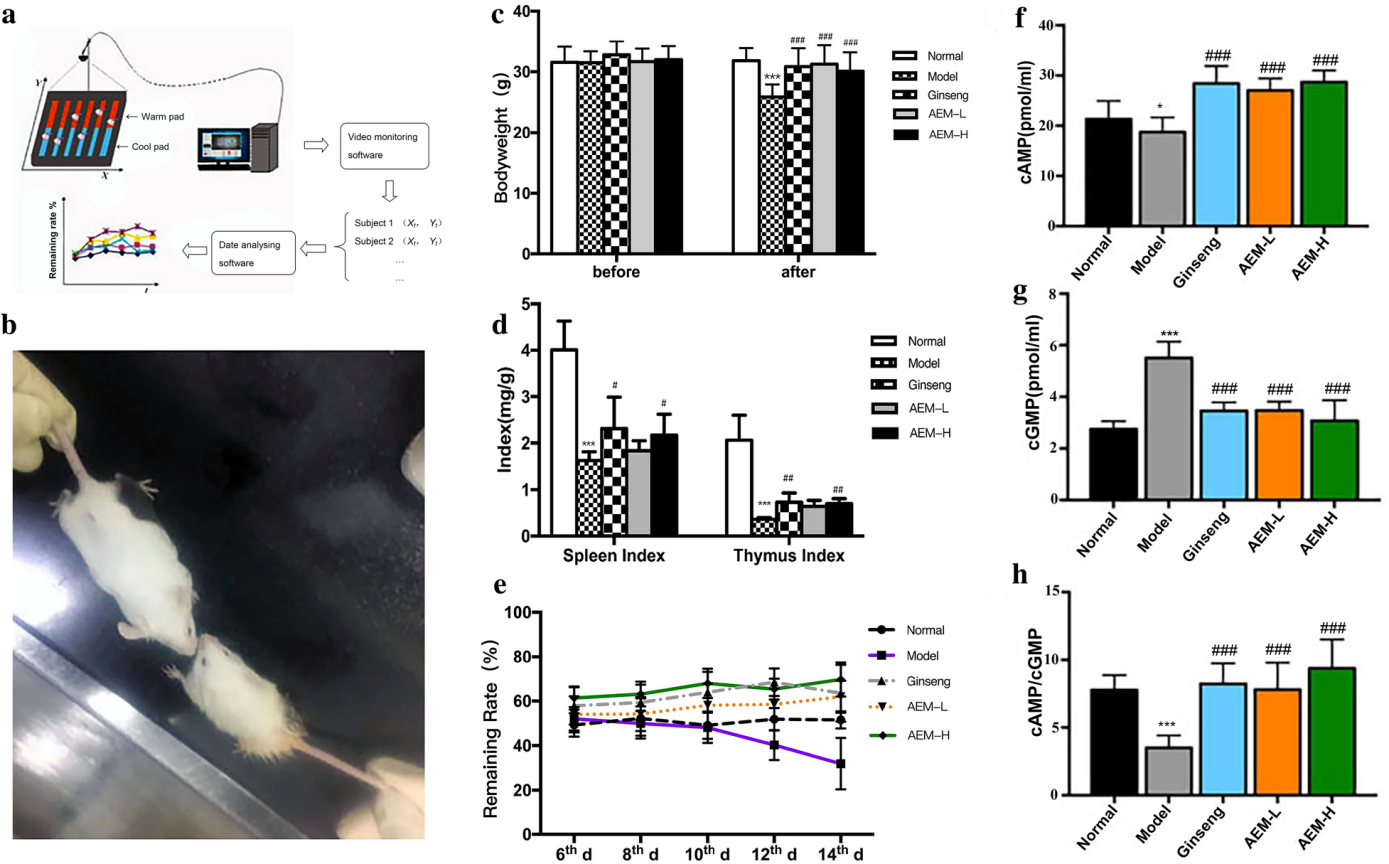
General conditions of mice. **a** The Animal Thermotropism Behavior Surveillance was consisted of automatic temperature controlling unit, remote monitoring unit, and data processing unit; **b** the left was normal mice and the right was model mice; **c** bodyweight of mice before and after induced by cyclophosphamide in five groups; **d** spleen and thymus indexes of mice in five groups; **e** changes in the temperature tropism of mice on cold/hot plates; **f**–**h** effect of Maca on the cAMP/cGMP ratio in serum. * represented *P* < 0.05 compared with the normal group; ** represented *P* < 0.01 compared with the normal group; *** represented *P* < 0.001 compared with the normal group; # represented *P* < 0.05 compared with the model group; ## represented *P* < 0.01 compared with the model group; ### represented *P* < 0.001 compared with the model group

The Animal Thermotropism Behavior Surveillance System was designed by China Military Institute of Chinese Materia Medica, 302 Military Hospital and was assembled by the Beijing Zhongjiao Instrument Company (Patent No: ZL2008200004444.2). The system consisted of automatic temperature controlling unit, remote monitoring unit, and data processing unit (Fig. 1a). The temperatures were maintained at 20.0 °C (cool plate) and 40.0 °C (warm plate), with a laboratory temperature of 23.0 °C ± 2.0 °C.

Half an hour after administration of AEM and ginseng, the animals were put on the cold/hot plate. Six mice were individually put into 6 monitoring channels one at a time, and could move freely across the two zones. We remotely monitored and recorded the temperature tropism behaviors and trajectories of each mouse. Records were taken once every day, 20 min each time, for 5 days in succession (2 days before the model set, and 3 days after the model set). Remaining rate (RR) was calculated as time remaining on the cold plate (s)/the total monitoring time (s) × 100%.

### Serum cAMP/cGMP

Blood was collected in clean test tubes with or without ethylenediaminetetraacetic acid (EDTA) following eyeball extraction. Serum was prepared by centrifugation at 4 °C and 1000×g for 10 min. The concentrations of cAMP and cGMP were measured by radioimmunoassay using assay kit (Sino-UK, Beijing, China).

### Biochemical assays of energy metabolism

After killing, the livers were excised immediately and immersed in − 80 °C liquid nitrogen. The liver homogenates were prepared in 0.1 g mL^−1^ wet weight of ice-cold isotonic physiological saline. The samples were centrifuged at 1000 rpm for 5 min and the supernatant was used to measure hepatic glycogen (HG) and LDH activities using assay kits (Jiancheng, Nanjing, China). The liver homogenates were also used for measuring Na^+^–K^+^-ATPase and Ca^2+^–Mg^2+^-ATPase activities. We counted 1 μmol of inorganic phosphorus, produced by ATPase in 1 mg of tissue protein within 1 h (μmol mg^−1^ h^−1^).

### Hematological parameters

Total 200 μl blood with EDTA (50 μl) was used for peripheral hemogram analysis by Beckman Coulter and T5 full-automatic blood cell analyzer. Blood samples were collected for detection of white blood cells (WBC), red blood cells (RBC), hemoglobin (HGB), and platelets (PLT).

### Measurement of spleen cytokines expression

Total RNA was isolated using Trizol (Invitrogen, Carlsbad, USA), and was reverse-transcribed into cDNA using a Super RT cDNA kit. The SYBR green Realtime PCR Master Mix was used for RT-qPCR amplification. The primers used for PCR are shown in Table 1. The cycling conditions were 95 °C for 10 min, followed by 40 cycles of 60 °C for 15 s, 75 °C for 60 s, and 95 °C. Temperature increases were 1 °C per 20 s. The RT-qPCR analysis was performed with the Light Cycler 480 RT-qPCR System (Roche, Basel, Switzerland). Fold-changes in gene expression were estimated using the CT comparative method normalizing β-actin CT values and relative to control samples as follows: ΔCt = Ct (assayed samples)-Ct(β-actin); ΔΔCt = ΔCt−ΔCt control; fold difference = 2^−(ΔΔCT)^.

**Table 1:** Primers used for quantitative RT-PCR

Genes	Forward (5′–3′)	Reverse (5′–3′)
β-actin	GGCTGTATTCCCCTCCATCG	CCAGTTGGTAACAATGCCATGT
IL-2	TGAGCAGGATGGAGAATTACAGG	GTCCAAGTTCATCTTCTAGGCAC
IFN-γ	ATGAACGCTACACACTGCATC	CCATCCTTTTGCCAGTTCCTC
TNF-β	GTCTGTGTATCCGGGACTTCA	TCTCCCTTACTGAGCAGGAAC
IL-4	CCCCAGCTAGTTGTCATCCTG	CAAGTGATTTTTGTCGCATCCG

### Western blot analysis and ELISA assays

The total protein in each spleen was extracted with lysis buffer, and 50 mg protein was resolved on a 10% sodium dodecyl sulfate polyacrylamide gel. The fractionated proteins were electrophoretically transferred to an immobilon polyvinylidene difluoride membrane and probed with antibodies of IFN-γ, TNF-β, IL-2 and IL-4 (Bioss. Inc., Beijing, China). The serum IFN-γ, TNF-β, IL-2 and IL-4 was quantified by using a commercially available ELISA kit (eBioscience) according to the manufacturer’s instructions.

### Statistical analysis

SPSS 17.0 was used for statistical analysis. All data were expressed as mean ± SD. Statistical analysis was performed using one-way analysis of variance, followed by the least-significant difference (LSD) post hoc test or Dunnett T3 test for comparison of multiple groups. Statistical significance was considered at *P *< 0.05.

## Results

### General conditions of mice

Compared with the normal animals, CYP-induced (spleen-deficient) mice exhibited reduced food intake, stagnant growth, less motion, disheveled fur, cuddled up with each other, and slouching (Fig. 1b). Nevertheless, the mice administrated with AEM and ginseng were normal, similar with the normal group.

Before the injection of CYP, the bodyweight of ginseng group, AEM-L group and AEM-H group did not significantly change, compared with the normal group (Fig. 1c). After injection of CYP, the bodyweights of model mice were significantly decreased ( < 0.001) (Fig. 1c). However, bodyweights of mice in the ginseng, AEM-L and AEM-H groups showed no significant declines. It was significantly different with that of the model group ( < 0.001) (Fig. 1c).

Thymus and spleen indices in model mice were significantly decreased after CYP treatment (Fig. 1d,  < 0.001). The AEM-H and ginseng groups exhibited significantly increased spleen ( < 0.001) and thymus indices ( < 0.01). Moreover, administration of AEM at 750 and 1500 mg/kg significantly (*P* < 0.05) enhanced bodyweight and organ indices of the spleen and thymus. AEM reversed loss of bodyweight and immune organ atrophy (spleen and thymus). CYP treatment drastically reduced the bodyweight and the immune organ indices of spleen and thymus.

### Changes in the temperature tropism of mice on cold/hot plate

We measured the RR on cool plate for 5 days, 3 days before injection of CYP (days 6, 8, and 10) and 2 days (days 12 and 14) after that. As shown in Fig. 1e, the cool plate RR was about 50% in the normal group. Compared with the normal group, the cool plate RR significantly increased for both AEM (*P *< 0.01) and ginseng groups (*P *< 0.001) before injection of CYP (Table 2). After the CYP injection, compared with the normal group, the RR on the cool plate in model group significantly decreased (*P *< 0.001). Both ginseng and AEM groups showed modest reductions in RR, which was significantly different with the model group (*P *< 0.001, Table 2). The increased RR on cool plate of ginseng and AEM groups manifested as “cold” tropism, suggesting an enhancement of “hot” production when ginseng and AEM doses were given. In contrast, decreased RR of model group manifested as “hot” tropism, suggesting a “cold” model by CYP treatment of the mice.

**Table 2:** Changes in the temperature tropism of mice on cold/hot plate

Groups	6th day	8th day	10th day	12th day	14th day
Normal	49.29 ± 5.21	52.20 ± 8.97	49.15 ± 6.10	51.84 ± 5.03	51.51 ± 3.84
Model	52.03 ± 5.26	49.97 ± 5.54	48.10 ± 6.88	40.27 ± 6.72^***^	31.90 ± 11.56^***^
Ginseng	57.84 ± 8.37^**^	59.38 ± 7.96^*^	63.96 ± 9.16^***^	68.46 ± 6.14^c^	63.55 ± 12.75^c^
AEM-L	54.04 ± 8.06	54.17 ± 7.63	58.10 ± 9.71^**^	58.53 ± 6.98^c^	61.96 ± 7.13^c^
AEM-H	61.39 ± 5.19^***^	63.16 ± 5.54^**^	67.97 ± 6.58^***^	69.68 ± 7.60^c^	65.35 ± 4.68^c^

^a^ Represented *P* < 0.05 compared with the model group; ^b^ represented *P* < 0.01 compared with the model group; ^c^ represented *P* < 0.001 compared with the model group

* Represented *P* < 0.05 compared with the normal group; ** represented *P* < 0.01 compared with the normal group; *** represented *P* < 0.001 compared with the normal group

### Effect of Maca on cAMP/cGMP in serum

As shown in Fig. 1f–h, compared with the normal group, cAMP/cGMP was significantly decreased in the model group (*P *< 0.001). Compared with the model group, the ratio of cAMP/cGMP significantly increased after the intake of ginseng (*P *< 0.05) and Maca powder (*P *< 0.001), with a low dose of 0.75 g/kg and a high dose of 1.5 g/kg, respectively.

### Effects of Maca on energy metabolism and hematological parameters

**Fig. 2:**
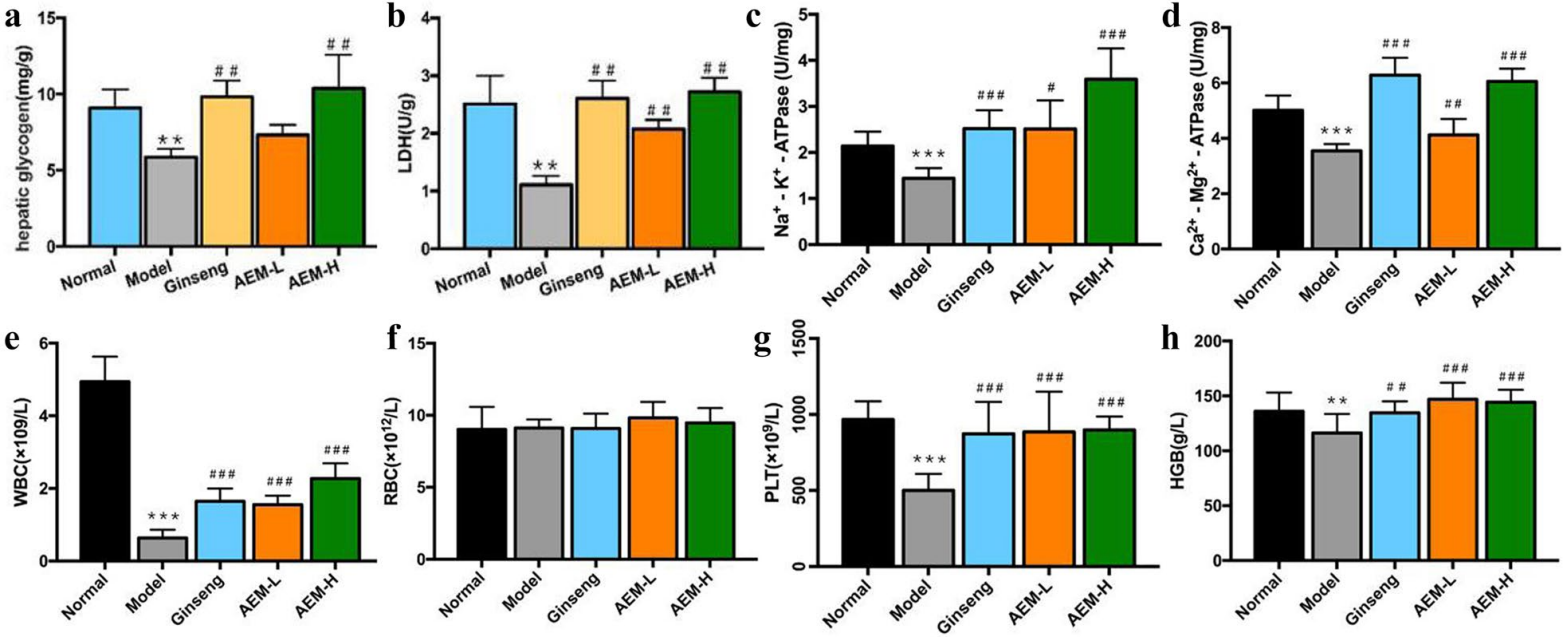
Effects of Maca on energy metabolism and hematological parameters. **a**, **b** Effect of Maca on the hepatic glycogen (**a**) and lactate dehydrogenase (LDH, **b**); c-d effect of Maca on the activities of Na^+^–K^+^-ATPase (**c**) and Ca^2+^–Mg^2+^-ATPase (**d**) in liver tissue; **e**–**h** effects of Maca on hematological parameters, including WBC, RBC, platelet (PLT) and HGB. * represented *P* < 0.05 compared with the normal group; ** represented *P* < 0.01 compared with the normal group; *** represented *P* < 0.001 compared with the normal group; # represented *P* < 0.05 compared with the model group; ## represented *P* < 0.01 compared with the model group; ### represented *P* < 0.001 compared with the model group

As shown in Fig. 2a, compared with control group, the HG level in liver homogenates was decreased in mice injected with CYP (*P *< 0.001). Compared with model group, the levels of HG were significantly increased in both the ginseng and AEM-H groups (*P *< 0.001), but there was no change in the AEM-L group. As shown in Fig. 2b, the liver level of LDH in the model group significantly decreased (*P *< 0.001) compared with the control group. However, the levels of LDH in the ginseng (*P *< 0.01) and Maca groups were increased (*P *< 0.001). As shown in Fig. 2c, d, the activities of Na^+^–K^+^-ATPase and Ca^2+^–Mg^2+^-ATPase significantly decreased after treated with CYP (*P *< 0.001), but the levels of Na^+^–K^+^-ATPase and Ca^2+^–Mg^2+^-ATPase significantly increased in both ginseng (*P *< 0.01) and AEM-H groups (*P *< 0.001). There was no change in the AEM-L group.

As shown in Fig. 2e, f, WBC and RBC counts decreased significantly following injection of CYP, compared with the control group (*P *< 0.001). However, the ginseng and AEM-H groups exhibited significantly increased WBC counts compared with the model group (*P *< 0.001), without return to normal levels. There were no significant changes in RBC counts. CYP significantly reduced the platelet counts (*P *< 0.05) and HGB (*P *< 0.001) content compared to the control group. The AEM-H group had significantly increased HGB compared to the model group (*P *< 0.05), but there were no changes in platelet counts (Fig. 2g, h).

### Effects of Maca on spleen cytokines of IFN-γ, TNG-β, IL-2, IL-4, T-bet and GATA-3

**Fig. 3:**
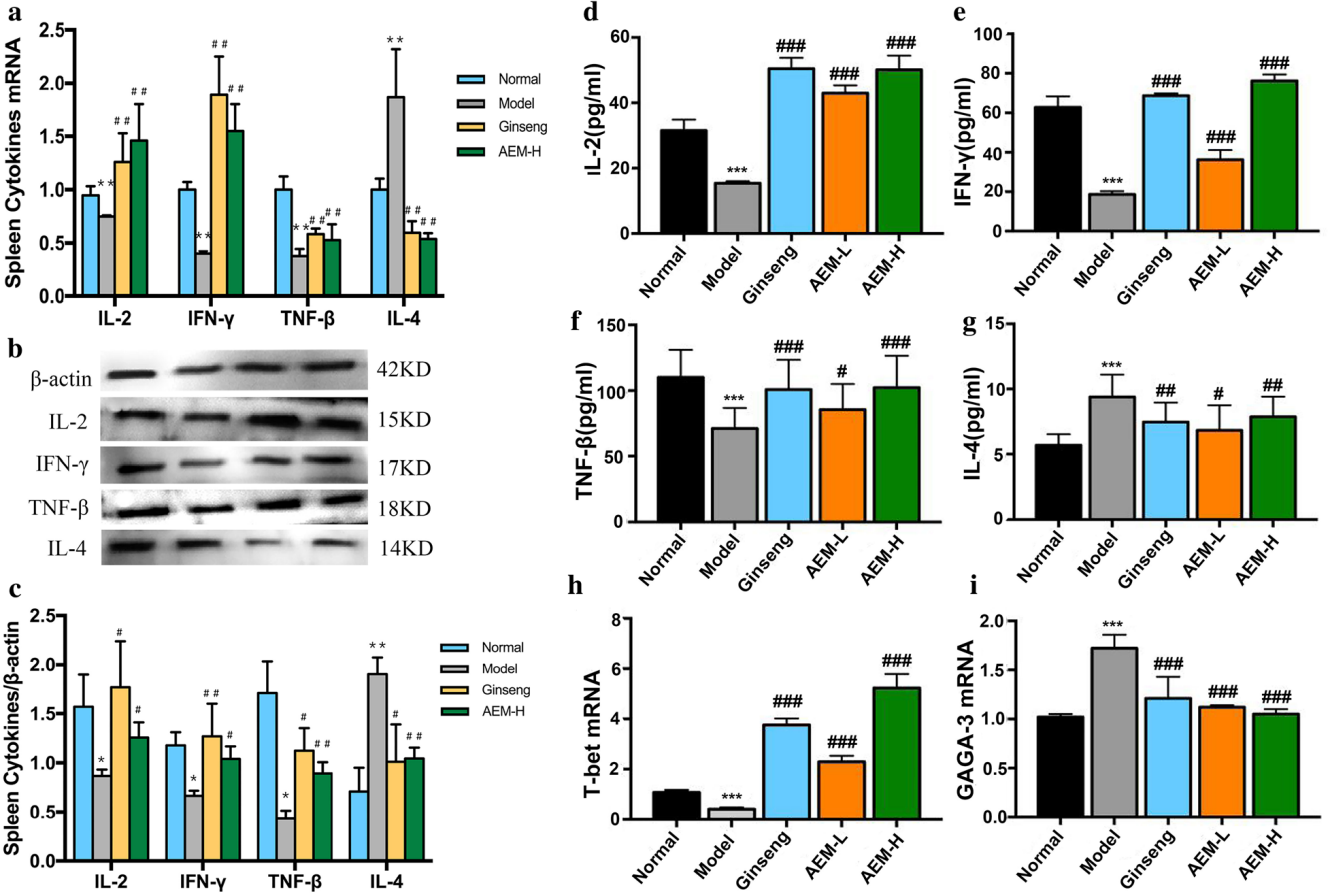
Effects of Maca on cytokines of IFN-γ, TNG-β, IL-2, IL-4. **a**, **h**, **i** Spleen cytokines of IFN-γ, TNG-β, IL-2, IL-4, T-bet and GATA-3 at mRNA level; **b**, **c** spleen cytokines of IFN-γ, TNG-β, IL-2, IL-4 at protein levels; **d**–**g** serum IFN-γ, TNG-β, IL-2, IL-4 level in each group; at mRNA and protein levels. * represented *P* < 0.05 compared with the normal group; ** represented *P* < 0.01 compared with the normal group; *** represented *P* < 0.001 compared with the normal group; # represented *P* < 0.05 compared with the model group; ## represented *P* < 0.01 compared with the model group; ### represented *P* < 0.001 compared with the model group

As shown in Fig. 3a–g and Table 3, we examined the expression of spleen cytokines of IFN-γ, TNG-β, IL-2, and IL-4 at mRNA and protein levels. Our results showed that Maca powder significantly improved the expression of IFN-γ, TNF-α, IL-2, and decreased IL-4 both at mRNA and protein levels in spleen and serum (*P* < 0.05 or *P* < 0.01 or *P* < 0.001). The impact of Maca and ginseng on spleen cytokines of IFN-γ, TNG-β, IL-2, and IL-4 was similar. The results showed that IFN-γ, TNF-β and IL-2 belonged to the positive factors and were secreted by Th1 cell. IL-4 was a negative factor and secreted by Th2 cell. We examined the expression of T-bet and GATA-3 at mRNA level (h-i). Maca powder significantly improved the expression of T-bet, and decreased GATA-3 (*P* < 0.05 or *P* < 0.01 or *P* < 0.001). Therefore, the imbalance of T-bet/GATA-3 mRNA level was closely related to Th1/Th2 imbalance and could be used as a new indicator to measure Th1/Th2 balance.

**Table 3:** Serum IFN-γ, TNG-β, IL-2, IL-4 level in each group

Groups	IL-2 (pg/ml)	IFN-l (pg/ml)	TNF-Nl (pg/ml)	IL-4 (pg/ml)
Normal	39.42 ± 7.22	69.20 ± 11.64	110.02 ± 21.07	5.69 ± 0.84
Model	20.31 ± 5.19^***^	30.63 ± 7.35^***^	71.11 ± 15.65^***^	9.39 ± 1.71^***^
Ginseng	48.04 ± 9.72^c^	58.49 ± 9.25^c^	100.78 ± 22.65^c^	7.46 ± 1.49^b^
AEM-L	50.84 ± 10.63^c^	60.33 ± 10.29^c^	85.42 ± 19.63^a^	6.83 ± 1.55^a^
AEM-H	56.73 ± 11.62^c^	75.39 ± 13.70^c^	102.30 ± 24.30^c^	7.87 ± 1.92^b^

^a^ Represented *P* < 0.05 compared with the model group; ^b^ represented *P* < 0.01 compared with the model group; ^c^ represented *P* < 0.001 compared with the model group

* Represented *P* < 0.05 compared with the normal group; ** represented *P* < 0.01 compared with the normal group; *** represented *P* < 0.001 compared with the normal group

## Discussion

In TCM, the medicinal properties of cold, cool, warm, and hot are associated with energy metabolism [12, 15].  After CYP treatment, the cool plate RR was found to be significantly decreased. Therefore, the CYP model was a Cold Zheng model, which had a state of deficiency and cold, showing obvious characteristics of “evil cold and like warmth”, and have spleen-deficiency properties [16]. After administered ginseng and Maca, energy transfer occurred, and the animals’ bodies became significantly warmer, increasing the RR on the cool plate. This indicates that the “cold” tropism was significantly enhanced by administration of ginseng and Maca, causing the mice to present a compensatory “cold” tropism. The results indicated that Maca exhibited the warm property, which can help to enhance the activity of energy metabolizing enzymes, and promote the internal mechanism of energy metabolism of body. Therefore, Maca has the characteristics of strengthening the spleen similarly with ginseng [3, 6, 8, 17]. Ginseng possesses the warm property, and can alleviate physical fatigue by strengthening the spleen [11]. We hypothesized that, similar to ginseng, Maca had properties of warm and strengthening of the spleen [18].

The cAMP and cGMP are objective indicators of Yin and Yang and may be the respective causes of Yin and Yang syndrome. When cAMP levels are reduced and cGMP levels are increased, the symptoms of Yang-deficiency emerge [10, 12]. On the other hand, as the ratio of cAMP/cGMP increases, symptoms of Yin-deficiency appear. As the second messenger of cell regulation, cAMP and cGMP act as intermediaries of hormones, neural mediators, and drugs. The cAMP/cGMP ratio of mice in the model group was significantly reduced. This verified the cold status of the CYP-induced spleen-deficiency syndrome model by biochemical indicators. Moreover, the ratio of cAMP/cGMP in both Maca groups increased, indicating that the TCM property of Maca is warm.

It was reported previously that cold or hot medicines could change the activities of ATPase, and this phenomenon was due to drug effects on energy metabolism [8]. In this study, we found that the increases of Na^+^–K^+^-ATPase and Ca^2+^–Mg^2+^-ATPase activities in both Maca and ginseng groups. It showed that heat production increased in Maca group was similar to the ginseng group. This indicated that changes in energy metabolism correlated with the “cold” or “hot” properties of herbal medicines. Increased ATPase activities in liver tissues may be one mechanism by which Maca increases heat production. The energy metabolism results obtained herein indicated that Maca had the hot/warm property of TCM [10, 19, 20]. TCM property of Maca was determined to be warm, different from hot to some extent.

Related studies have reported that SDS might change the course of HG and LDH, which were related to fatigue. The mechanisms behind physical fatigue may include consumption of glycogen and the systemic accumulation of metabolic products, including lactic acid and ammonia [8, 10]. The HG in the AEM-H group increased significantly and the level of LDH increased in both the AEM-H and AEM-L groups. Maca powder could relieve bodily fatigue, particularly at a dose of 1.5 g/kg (about 9 g/kg for a person per day). Therefore, Maca may be able to ameliorate spleen-deficiency syndrome.

The body weight in model group was decreased, while this decrease was reversed in mice within both Maca and ginseng groups. This suggests that Maca plays a positive role in preventing body weight loss that results from CYP. Thymus and spleen indices are frequently used as hallmarks for evaluating the success of immunosuppression animal models.^17^ Loss of stem cells and the inability of bone marrow to regenerate new blood cells results in thrombocytopenia and leukopenia caused by CYP, potentially leading to significant morbidity and mortality. Peripheral blood cell counts directly reflect the body’s immune system status. Our results showed that Maca significantly reversed declining WBC and platelet counts, and increased HGB.

T cells play an important role in the immune system and include T helper (Th) cells, T cytotoxic cells, T suppressor cells, and T effector cells [15]. The functions of these subsets of Th cells depend upon the specific types of cytokines that are generated, such as IL-2, IFN-γ, TNF-α and GM-CSF by Th1 cells, and IL-4, IL-5, IL-6 and IL-10 by Th2 cells. These cytokines can directly or indirectly regulate immune reactions. For example, IL-2 activates NK cells to decrease tumor masses and inhibit tumor cell metastases. CYP can rapidly lead to abrupt change in Th1/Th2 bias. Previous studies on the immunologic effect of CYP revealed a decrease in the absolute number of T cells, circulating B cells, a decrease in the secretion of IFN-γ, TNF-α, IL-2, and production of IL-4. In this study, our results showed that Maca powder improved the levels of IFN-γ, TNF-α, IL-2, and decreased IL-4 in plasma, and mRNA levels and protein levels in spleen. IFN-γ could promote the process that the Th0 cells differentiated into Th1 cell and inhibited Th2 cell proliferation, and IL-4 could induce Th0 cells to differentiate into Th2 cell and inhibit Th1 cell differentiation. Therefore, IFN-γ and IL-4 was a pair of important immune cytokines that antagonized each other. In contrast, GATA-3 could improve the transcription activity of the promoter of IL-4, inhibit the expression of IFN-γ, induce Th0 cells to differentiate into Th2 cells, and inhibit Th1 cell differentiation. Therefore, the imbalance of T-bet/GATA-3 mRNA level was closely related to Th1/Th2 imbalance and could be used as a new indicator to measure Th1/Th2 balance. This suggested that Maca may enhance cell-mediated and humoral immune responses by increasing the secretion of Th1 and decreasing Th2 cytokines and at the same time improve the proliferation and transformation of splenic T lymphocytes, showing that it can significantly improve the model’s low immune status. Therefore, Maca may improve the spleen deficiency in mice by improving immunity [21].

## Conclusion

Maca possesses the TCM property of warm and the function of spleen strengthening. Our findings provide experimental evidence to support further research and clinical application of Maca within TCM. Our work could also help generate kinds of TCM resources.

## Data Availability

The raw data were collected and analyzed by the authors, and are not ready to share their data because the data have not been published.
